# *In Vivo* Raman Spectroscopy Reveals Biochemical Composition of the Esophageal Tissue in Pediatric Eosinophilic Esophagitis

**DOI:** 10.14309/ctg.0000000000000665

**Published:** 2023-12-19

**Authors:** Andrea Locke, Ezekiel Haugen, Giju Thomas, Hernan Correa, Evan S. Dellon, Anita Mahadevan-Jansen, Girish Hiremath

**Affiliations:** 1 Department of Biomedical Engineering, Vanderbilt University, Nashville, Tennessee, USA;; 2 Vanderbilt Biophotonics Center, Nashville, Tennessee, USA;; 3 Department of Chemistry, Vanderbilt University, Nashville, Tennessee, USA;; 4 Division of Pediatric Pathology, Monroe Carell Jr. Children's Hospital at Vanderbilt, Vanderbilt University Medical Center, Nashville Tennessee, USA;; 5 Division of Gastroenterology and Hepatology, University of North Carolina, Chapel Hill, North Carolina, USA;; 6 Division of Pediatric Gastroenterology, Hepatology, and Nutrition, Monroe Carell Jr. Children's Hospital at Vanderbilt, Vanderbilt University Medical Center, Nashville, Tennessee, USA.

**Keywords:** eosinophilic esophagitis, Raman spectroscopy, biochemical alterations, real-time assessment, spectral markers, diagnosis, monitoring

## Abstract

**INTRODUCTION:**

Biochemical alterations in the esophagus of patients with eosinophilic esophagitis (EoE) are poorly understood. We used Raman spectroscopy through a pediatric endoscope to identify key Raman features reflective of the esophageal biochemical composition to differentiate between children with EoE from non-EoE controls and between children with active (aEoE) and inactive EoE (iEoE).

**METHODS:**

Spectral measurements were obtained using a customized pediatric endoscope-compatible fiber-optic Raman probe in real time during an esophagogastroduodenoscopy. Chemometric analysis was performed to identify key Raman features associated with EoE. Pearson correlation analysis was used to assess relationship between the key Raman features and EoE activity indices. Their diagnostic utility was ascertained using the receiver operator characteristic curve analysis.

**RESULTS:**

Forty-three children were included in the study (EoE = 32 [74%] and non-EoE control = 11 [26%]; aEoE = 20 [63%] and iEoE = 12 [37%]). Raman intensities assigned to lipids, proteins, and glycogen:protein ratio accurately distinguished children with EoE from those without EoE and aEoE from iEoE. They significantly correlated with EoE activity indices. The Raman peak ratio for lipids had 90.6% sensitivity, 100% specificity, and an area under the curve of 0.95 to differentiate children with EoE from non-EoE controls. The glycogen:protein ratio had 70% sensitivity, 91.7% specificity, and an area under the curve of 0.75 to distinguish children with aEoE from iEoE.

**DISCUSSION:**

Real-time intraendoscopy Raman spectroscopy is an effective method for identifying spectral markers reflective of the esophageal biochemical composition in children with EoE. This technique may aid in the diagnosis and monitoring of EoE and help to elucidate EoE pathogenesis.

## INTRODUCTION

Eosinophilic esophagitis (EoE) is an increasingly prevalent allergen-mediated inflammatory condition of the esophagus. It is estimated to affect 1 in 2,000 individuals across all ages in Westernized countries (1). Children affected by this clinicopathologic disease typically present with feeding difficulties, vomiting, and abdominal pain, and their diagnosis is confirmed by an intense eosinophilic inflammation and accompanying architectural changes in the esophageal biopsies obtained during esophagogastroduodenoscopy (EGD) (2). A delay in diagnosing EoE or an inadequate control of the eosinophilic inflammation can lead to fibrostenotic complications such as esophageal narrowing and stricture requiring endoscopic interventions (3,4).

Light-based approaches such as optical coherence tomography and confocal laser endomicroscopy have been tried to facilitate diagnosis and investigate cellular and architectural changes in esophageal tissue affected by EoE (5–7). However, these approaches are not capable of profiling biochemical changes in tissue. Understanding the tissue-level biochemical alterations is critical in enhancing our understanding of the pathobiology of the disease process and improving the clinical outcomes because they precede the relevant architectural and cellular changes, influence the disease phenotype, and may predict treatment response (8–10).

Raman spectroscopy is a versatile light-based technique validated for profiling biochemical composition of a specimen rapidly at a very high spatial resolution with minimal sample preparation (11). It measures inelastic scattering when light interacts with various chemical bonds within a molecule. The generated spectra contain multiple peaks related to the vibrational modes of the chemical bonds within the sample making Raman spectroscopy uniquely suited for biomedical applications (12). For instance, the vibrational modes within lipids, proteins, nucleic acids, glycogens, and carbohydrates generate unique Raman peaks (13). Raman spectroscopy has been used to detect eosinophils in murine EoE models (14), and we have previously reported *in vitro* characterization of the biochemical composition of esophageal samples obtained from children with EoE (15). Furthermore, our group and others have also reported the application of endoscope-coupled Raman spectroscopy for *in vivo* characterization of pediatric- and adult-onset inflammatory bowel disease (16–19).

In this study, we investigated biochemical composition of the esophagus in a physiologic state (i.e., *in vivo*) during EGD using Raman spectroscopy. Our primary aim was to characterize and identify specific Raman intensities that distinguished children with EoE from those without EoE, as well as children with active EoE from inactive EoE using chemometric analytical techniques. Our secondary aims were to examine the relationship between Raman intensities in EoE and validated endoscopic and histologic EoE activity indices. We explored whether EoE-related biochemical alterations were evenly distributed across the esophagus and whether the distribution was influenced by disease activity. Based on our experiences with the application of endoscope-coupled Raman spectroscopy for characterization of inflammatory bowel disease in real time during endoscopy and *in vitro* analysis of esophageal samples from children with EoE, we hypothesized that *in vivo* esophageal Raman spectral features can be efficiently acquired in real time during EGD, and key Raman spectra can accurately distinguish children with EoE from those without EoE as well as children with active EoE from inactive EoE. Furthermore, we hypothesized that specific Raman intensities will correlate with the validated EoE activity indices.

## METHODS

### Study population and enrollment criteria

The study was conducted at Monroe Carell Jr. Children's Hospital at Vanderbilt University Medical Center between January 2019 and April 2021. Children between 6 and 17 years scheduled for EGD with biopsies for esophageal symptoms suggestive of EoE (such as vomiting, feeding difficulties, and abdominal pain) or with a previous diagnosis of EoE were invited to participate in the study. Those with known esophageal surgery, dysmotility, injury, stricture, varices, exposure to systemic corticosteroids within the past 30 days, and inflammatory bowel disease were excluded. The Vanderbilt University Medical Center Institutional Review Board approved this study (protocol #160785). Appropriate informed consent was obtained from the caregivers of the participants, and assent was obtained from the children before enrollment.

### Study groups

According to the 2018 AGREE consensus statement, children with symptoms of esophageal dysfunction and a peak eosinophil count (PEC) of ≥15 eosinophils per high-power field (eos/hpf) in the absence of other causes of esophageal eosinophilia were classified as having EoE. Based on their esophageal biopsy results and clinical presentation, children with EoE were further classified as having active EoE (aEoE) if their PEC was ≥15 eos/hpf or inactive EoE (iEoE) if their PEC was <15 eos/hpf (20). The non-EoE control group comprised children who underwent EGD with biopsies for upper gastrointestinal symptoms but did not have a previous diagnosis of EoE and did not meet the histologic criteria for EoE. The children in non-EoE control group had heterogeneous conditions including gastroesophageal reflux disease (GERD), functional dyspepsia, and irritable bowel syndrome.

### Clinical and endoscopic data

Demographic variables (such as age at EGD, sex, and ethnicity), clinical characteristics (such as presenting symptoms and previous diagnoses), atopic conditions (such as allergic rhinitis, eczema, and asthma), and medication exposure (such as exposure to proton-pump inhibitors and topical steroids) of the participants were collected prospectively at enrollment. G.H. performed all endoscopies/biopsies and scored the esophageal mucosal abnormalities per the validated Endoscopic Reference Scores (EREFS) (i.e., edema [0–2], rings [0–3], exudates [0–2], furrows [0–2], and strictures [0–1]). A higher EREFS score indicated greater endoscopic severity of EoE (21). G.H. had access to the clinical information but was blinded to histologic and Raman data.

### Histologic assessment

During EGD, 4–6 esophageal biopsies (2–3 each from the distal and proximal esophagus) were collected to maximize diagnostic sensitivity for clinical care. According to the standard protocol, biopsies were submitted for hematoxylin and eosin staining. The PEC and the degree of abnormality (grade) of the EoE-relevant epithelial features and lamina propria were assessed by H.C. per the histology scoring system (EoEHSS) (22) (see Supplementary Material, Supplementary Digital Content 1, http://links.lww.com/CTG/B49 for details about EoEHSS scoring). H.C. had access to clinical and endoscopic data but was blinded to Raman data.

### Portable Raman spectroscopy system and pediatric endoscope-compatible fiber-optic probe

We designed a portable Raman spectroscopic system and a customized pediatric endoscope-compatible fiber-optic Raman probe for seamless integration into the clinical workflow during EGD to collect *in vivo* Raman measurements (see Supplementary Figure 1, Supplementary Digital Content 2, http://links.lww.com/CTG/B50). The specifications of our portable Raman spectroscopic system are provided in Supplementary Material (see Supplementary Digital Content 1, http://links.lww.com/CTG/B49). The diameter of the Raman probe was 2.6 mm and could be easily advanced through the 2.8-mm biopsy channel in the endoscope. The system was controlled by a custom LabVIEW program capable of displaying the live preprocessed Raman spectra after excluding the background autofluorescence to prevent the collection of saturated signals and to ensure spectral quality.

### Acquisition of *in vivo* Raman spectra

With the endoscope in a stable position in the proximal esophagus, the Raman probe was advanced through the biopsy channel until the distal tip of the probe was directly visualized and gently placed on the luminal surface of the esophagus. Once in position, the white light was turned off to prevent interference with Raman spectra acquisition, and the near-infrared light was activated. Raman measurements were obtained from 2 to 3 spots in the proximal esophagus, and then, the process was repeated for the distal esophagus, with 3 Raman spectra collected per spot (Supplementary Video 1). Whenever possible, Raman spectra were acquired from the areas of mucosal abnormalities. Each Raman spectra were obtained using 80-mW power and 6 seconds of integration time (i.e., 2 seconds of exposure time and 3 spectral accumulations) per measurement. After the spectra were recorded, the remainder of the EGD with biopsies was completed. The safety and feasibility of this system has been previously reported (23).

### Data processing and statistical analysis

Descriptive statistics were used to characterize the cohort, including counts and percentages for categorical variables and median (range) for continuous variables. Acquired Raman spectra were preprocessed in MATLAB (Natick, MA). This preprocessing included noise smoothing using a second-order Savitzky-Golay filter, fluorescence background subtraction using a seventh-order polynomial fit, and mean normalization before averaging by spot for analysis (24,25). The Raman spectra from proximal and distal esophageal sites within the same patient were averaged together.

To identify key spectral differences between EoE and non-EoE controls and between aEoE and iEoE, we conducted principal component analysis (PCA) (26), followed by peak ratio analysis using PCA-identified Raman peaks. Descriptive statistics were used to compare the mean and median (interquartile range) for each study group. Pearson correlation (*r*) analyses examined the relationship between the key peak ratios and the EoE activity indices, including EREFS, PEC, and EoEHSS (grade). To determine the diagnostic value of the key peak ratios, feature ranking analysis was performed using the *P* value from a Kruskal–Wallis test to determine the peak ratio that substantially contributed toward the classification of EoE vs non-EoE control groups.

Next, receiver operating characteristic analysis was performed in MATLAB using a trained support vector machine classifier with leave-one-out cross-validation to evaluate the diagnostic utility (sensitivity, specificity, and the area under the curve [AUC]). Individual peak ratios associated with the lipid and protein content for each participant and their corresponding clinical diagnostic label were used to compute a receiver operating characteristic curve and identify threshold values that provided optimal sensitivity and specificity. Support vector machine and a “leave-one-out” cross-validation were then used to train a model on multiple peak ratios with a binary label for EoE (1) and non-EoE (0) and provide a probability for classification. The model determined the optimum thresholds for classification.

The same analysis plan was followed to address our exploratory aim, but the Raman spectra from the proximal and distal esophageal sites were compared against each other. OriginPro software (OriginLab, Northampton, MA) was used for statistical analyses. A conventional *P* value of <0.05 was used to determine statistical significance.

## RESULTS

### Cohort characteristics

A total of 43 children, including 32 (74%) with EoE and 11 (26%) non-EoE controls, participated in this study. The children with EoE were significantly younger than the non-EoE controls (median [interquartile range] 12 [9–15] vs 16 [15–17] years; *P* = 0.004). Consistent with the known epidemiology of EoE, 81% of children in the EoE group were male. However, all children (100%) in the non-EoE control group were female. More than 80% of patients in each group were White. A significantly lower proportion of children with EoE reported abdominal pain (6% vs 82%; *P* = 0.001) and reflux-like symptoms (3% vs 27%; *P* = 0.02) than non-EoE controls. As expected, EREFS, PEC, and EoEHSS (grade) were significantly higher in children with EoE than non-EoE controls. Within the EoE group, 20 (63%) had aEoE, and 12 (37%) had iEoE. Children with aEoE had significantly higher EREFS, PEC, and EoEHSS compared with iEoE (Table 1).

**Table 1.** Demographic, clinical, endoscopic, and histologic characteristics of the cohort

	Eosinophilic esophagitis (n = 32)			Controls (n = 11) (D)	*P* value	
	Overall (n = 32) (A)	Active EoE (n = 20) (B)	Inactive EoE (n = 12) (C)		A vs D	B vs C
Age, yr, median (IQR)	12 (9–15)	11 (10–13)	10 (8–13)	16 (15–17)	0.001	NS
Gender, n (%)						
Male	26 (81)	16 (80)	10 (83)	0 (0)	<0.001	NS
Female	6 (19)	4 (20)	2 (17)	11 (100)	<0.001	NS
Ethnicity, n (%)						
White	26 (81)	16 (80)	11 (92)	9 (82)	NS	NS
Black	4 (13)	3 (15)	1 (8)	1 (9)	NS	NS
Asian	1(3)	0(0)	0 (0)	0 (0)	NS	NS
Presenting symptom(s), n (%)						
Nausea	1 (3)	0 (0)	1 (8)	1 (9)	NS	NS
Vomiting	4 (13)	3 (15)	1 (8)	0 (0)	NS	NS
Feeding difficulties	6 (19)	4 (20)	2 (17)	1 (9)	NS	NS
Abdominal pain	2 (6)	1 (5)	1 (8)	9 (82)	<0.001	NS
Reflux-like symptoms	1 (3)	1 (5)	0 (0)	3 (27)	0.02	NS
Atopic comorbidities, n (%)						
Food allergies	13 (41)	9 (45)	4 (33)	2 (18)	NS	NS
Asthma	12 (38)	7 (35)	5 (42)	1 (9)	NS	NS
Eczema	10 (31)	4 (20)	6 (50)	0 (0)	0.04	NS
Allergic rhinitis	17 (53)	11 (55)	6 (50)	1 (9)	0.01	NS
Medication exposure(s), n (%)						
PPI	8 (25)	6 (30)	2 (17)	2 (9)	NS	NS
Topical steroids	3 (9)	2 (10)	1 (8)	0 (0)	NS	NS
Dietary elimination	3 (9)	2 (10)	1 (8)	0 (0)	NS	NS
Combination	16 (50)	8 (40)	8 (67)	0 (0)	0.004	NS
EREFS, median (IQR)	1 (0–2)	2 (1–2)	0 (0–0)	0 (0–0)	0.003	<0.001
PEC (eos/hpf), median (IQR)	38 (3–74)	74 (48–81)	0 (0–4)	0 (0–0)	<0.001	<0.001
EoEHSS (grade), median (IQR)	9 (4–14)	13 (10–17)	4 (4–5)	4(2–4)	<0.001	<0.001

EoE, eosinophilic esophagitis; EoEHSS, EoE Histology Scoring System; eos/hpf, eosinophils per high-power field; EREFS, Endoscopic Scoring System; IQR, interquartile range; NS, not significant; PEC, peak eosinophil count; PPI, proton-pump inhibitor.

### Key spectral features distinguish children with EoE from non-EoE control children and children with active EoE from children with inactive EoE

**Figure 1.**
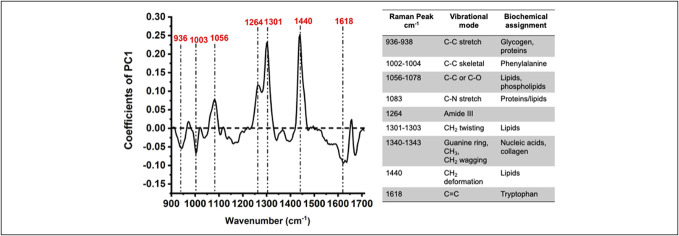
Loading plot from principal component analysis of Raman spectra highlights biochemical variance between children with EoE and non-EoE controls. The accompanying table provides the biochemical assignment for the differentiating Raman peaks. EoE, eosinophilic esophagitis.

The PCA identified that Raman peaks assigned to glycogen and proteins (936–938 cm^−1^), phenylalanine (1,002–1,004 cm^−1^), lipids and phospholipids (1,056–1,078 cm^−1^), proteins/lipids (1,083 cm^−1^), amide III (1,264 cm^−1^), lipids (1,301–1,303 cm^−1^), nucleic acids and collagen (1,340–1,343 cm^−1^), lipids (1,440 cm^−1^), and tryptophan (1,618 cm^−1^) distinguished EoE from non-EoE controls (Figure 1).

**Figure 2.**
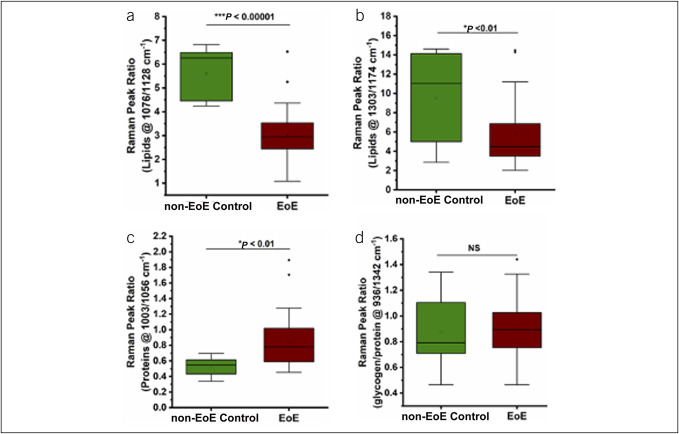
Raman peak ratios at (**a**) ∼1,076/1,128 cm^−1^ and (**b**) 1,303/1,174 cm^−1^ indicate significantly lower lipid content for EoE compared with non-EoE controls. Protein peak ratios at (**c**) ∼1,003/1,056 cm^−1^ and glycogen:protein ratio (**d**) 936/1,342 cm^−1^ indicate significantly higher protein content for EoE compared with non-EoE controls. EoE, eosinophilic esophagitis.

The lipid content at 1,076/1,128 cm^−1^ and 1,303/1,174 cm^−1^ was significantly lower in children with EoE (2.9 [2.4–3.5] and 4.5 [3.5–6.7], respectively) compared with non-EoE controls (6.3 [4.5–6.5] and 11.0 [5.5–13.4]; *P* < 0.00001 and *P* < 0.01, respectively). By contrast, the protein content (at 1,003/1,056 cm^−1^) was notably higher in children with EoE (0.78 [0.6–1.0]) compared with non-EoE controls (0.56 [0.4–0.6]; *P* < 0.01). The glycogen:protein ratio (936/1,342 cm^−1^) was comparable between children with EoE (0.9 [0.8–1.0]) and non-EoE controls (0.8 [0.7–1.1]; *P* = 0.65) (Figure 2).

Next, we examined whether these differences were related to the EoE activity status. We observed that the lipid content (at 1,076/1,128 cm^−1^ and 1,303/1,174 cm^−1^) and the protein content (at 1,003/1,056 cm^−1^) were comparable between children with aEoE and iEoE. Children with aEoE had significantly lower glycogen:protein ratio (936/1,342 cm^−1^) (0.8 [0.7–1.0]) compared with children with iEoE (1.0 [0.9–1.1]; *P* < 0.01) (see Supplementary Figure 2, Supplementary Digital Content 3, http://links.lww.com/CTG/B51).

### Raman peak ratios for lipid and protein content correlate with EoE activity indices

We observed a negative correlation between the Raman peak ratios reflective of lipid content and EREFS (for 1,076/1,128 cm^−1^: −0.42, *P* = 0.008; for 1,303/1,174 cm^−1^: *r* = −0.38, *P* = 0.01), PEC (for 1,076/1,128 cm^−1^: *r* = −0.37, *P* = 0.02), and EoEHSS score (for 1,076/1,128 cm^−1^: *r* = −0.40, *P* = 0.01; for 1,303/1,174 cm^−1^: *r* = −0.36, *P* = 0.02). Only the lipid content at 1,303/1,174 cm^−1^ did not correlate with PEC. The protein content (at 1,003/1,056 cm^−1^) positively correlated with EREFS (*r* = 0.48, *P* = 0.002), PEC (*r* = 0.35, *P* = 0.02), and EoEHSS score (*r* = 0.47, *P* = 0.001). However, the glycogen:protein ratio (at 936/1,342 cm^−1^) did not significantly correlate with any of the EoE activity indices (EREFS [*r* = 0.28, *P* = 0.09], PEC [*r* = −0.22, *P* = 0.06], and EoEHSS score [*r* = −0.28, *P* = 0.18]) (Table 2).

**Table 2.** Pearson correlation between the key Raman peak ratios and the EoE activity indices

	Key Raman peaks/peak ratio			
	Lipids (1,076/1,128 cm^−1^)	Lipids (1,303/1,174 cm^−1^)	Protein (1,003/1,056 cm^−1^)	Glycogen:protein ratio (936/1,342 cm^−1^)
EREFS	−0.42**	−0.38*	0.48**	−0.28
PEC	−0.37*	−0.30	0.35*	−0.31
EoEHSS (grade)	−0.43**	−0.33*	0.47**	−0.28

EoE, eosinophilic esophagitis; EoEHSS, EoE Histology Scoring System; EREFS, Endoscopic Scoring System; PEC, peak eosinophil count.

*P < 0.05, **P < 0.01.

### Raman features accurately distinguish EoE and its activity status

**Figure 3.**
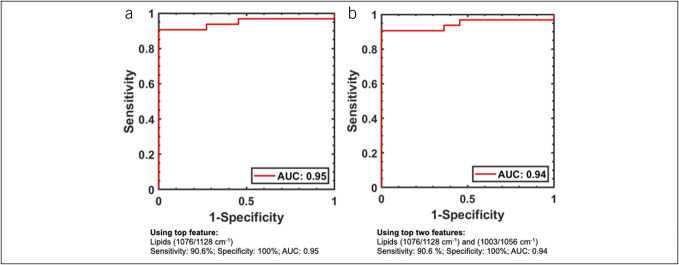
Receiver operating characteristics (support vector machine classification model) to determine the diagnostic accuracy of Raman peak ratios (**a**) 1,076/1,128 cm^−1^ and (**b**) at 1,076/1,128 cm^−1^ and 1,003/1,056 cm^−1^ combined, reflective of lipid and protein contents, respectively, in differentiating EoE from non-EoE controls. EoE, eosinophilic esophagitis.

The feature ranking analysis revealed that the Raman peaks assigned to lipid content at 1,076/1,128 cm^−1^ and protein at 1,003/1,056 cm^−1^ were the top 2 features for classification, and of these, the peak ratio at 1,076/1,128 cm^−1^ had the highest importance score. The feature ranking analysis identified the glycogen:protein ratio (at 936/1,342 cm^−1^) with the highest importance score to distinguish between children with aEoE from children with iEoE (see Supplementary Figure 3, Supplementary Digital Content 4, http://links.lww.com/CTG/B52). The Raman peak ratio at 1,076/1,128 cm^−1^ at a threshold of 0.75 had sensitivity of 90.6%, specificity of 100%, with an AUC of 0.95, and the combined peak ratios (1,076/1,128 cm^−1^ and 1,003/1,056 cm^−1^) at a threshold of 0.72 also had a sensitivity of 90.6%, specificity of 100%, with an AUC of 0.94 to differentiate children with EoE from non-EoE controls (Figure 3). The glycogen:protein ratio (at 936/1,342 cm^−1^) had a sensitivity of 70%, specificity of 91.7%, and an AUC of 0.77 to distinguish children with aEoE from iEoE.

On comparing the performance of Raman peak ratios and EREFS, Raman peak ratios had substantially higher sensitivity (lipid content [1,076/1,128 cm^−1^]: 90.6% and lipid + proteins [1,076/1,128 cm^−1^ + 1,003/1,056 cm^−1^]: 90.6%), specificity (lipid content [1,076/1,128 cm^−1^]: 100% and lipid + proteins [1,076/1,128 cm^−1^ + 1,003/1,056 cm^−1^]: 100%), and AUC (lipid content [1,076/1,128 cm^−1^]: 0.95 and lipid + proteins [1,076/1,128 cm^−1^ + 1,003/1,056 cm^−1^]: 0.94) than EREFS (sensitivity: 50%, specificity: 50%, and AUC 0.5) in differentiating EoE from non-EoE controls. When differentiating aEoE from iEoE, Raman peak ratios for glycogen:protein (936/1,342 cm^−1^) had modest sensitivity (70%), high specificity (91.7%), and modest AUC (0.75), whereas EREFS had high sensitivity (94.4%), specificity (100%), and AUC (0.97).

### Raman intensities differ between proximal and distal esophagus in children with active EoE

In children with aEoE, the lipid (at 1,303/1,174 cm^−1^) was significantly higher in proximal esophagus compared with distal esophagus, and the protein content (at 1,003/1,056 cm^−1^) and the glycogen:protein ratio (at 936/1,342 cm^−1^) were significantly lower in distal esophagus compared with proximal esophagus. By contrast, the lipid content (at 1,076/1,128 cm^−1^ and 1,303/1,174 cm^−1^), protein content (at 1,003/1,056 cm^−1^), and the glycogen:protein ratio (at 936/1,342 cm^−1^) comparable across the proximal and distal esophagus in children with iEoE (see Supplementary Table 1, Supplementary Digital Content 5, http://links.lww.com/CTG/B53).

## DISCUSSION

Substantial advances have been made elucidating the environmental (27), genetic and epigenetic (28), and molecular and immunological basis of EoE (29,30). However, little is known about the biochemical changes in the esophageal tissue affected by this debilitating disease (31). We developed a customized pediatric endoscope-compatible fiber-optic Raman probe to profile esophageal biochemical composition in real time during EGD and found that the Raman intensities assigned to lipids (1,076/1,128 cm^−1^ and 1,303/1,174 cm^−1^), proteins (1,003/1,056 cm^−1^), and glycogen:protein ratio (at 936/1,342 cm^−1^) distinguished children with EoE from those without EoE and correlated with validated metrics of endoscopic and histologic EoE activity. Furthermore, Raman markers for lipids and proteins (1,076/1,128 cm^−1^ and 1,003/1,056 cm^−1^, respectively) differentiated children with EoE from non-EoE controls, and the glycogen:protein ratio (at 936/1,342 cm^−1^) differentiated children with aEoE from those with iEoE with high sensitivity, specificity, and accuracy. These findings add to our understanding of the pathobiology of EoE and lay the foundation for a highly sensitive biopsy independent approach to identify EoE with biochemical specificity in real time during EGD.

Healthy esophageal tissue contains abundant mature cells in the epithelial layer with a balanced composition of glycogen and protein content. The mature cells have phospholipids in the cell membranes, lipid droplets in the cytoplasm, and lipid-based cellular structures and large adipose cells in the submucosal layer (32). During an inflammatory process, such as in EoE, cytokines and chemokines disrupt the squamous epithelium. The accompanying hypermetabolic state results in varying degrees of basal zone hyperplasia and a higher proportion of immature and maturing squamous cells (33,34). These changes are reflected in an altered biochemical composition of the esophageal tissue, with increased protein and decreased lipid and glycogen content in active EoE and advance our understanding of the disease pathobiology.

Our previous *in vitro* study demonstrated that the peak intensities associated with glycogen (at 936/1,449 cm^−1^), protein (at 1,660/1,209 cm^−1^), and lipid (at 1,301/1,260 cm^−1^) content were able to distinguish between esophageal biopsies obtained from children with EoE from non-EoE controls and differentiate between esophageal biopsies from children with active and inactive EoE. In this study, Raman intensities also strongly correlated with EREFS, PEC, and EoEHSS (grade). We further validated these findings by performing Raman mapping on an independent set of esophageal samples (15). Our current *in vivo* study found that different key Raman intensities reflecting lipids (1,076/1,128 cm^−1^ and 1,303/1,174 cm^−1^), protein (1,003/1,056 cm^−1^), and glycogen:protein ratio (at 936/1,342 cm^−1^) could identify children with EoE and differentiate them based on their disease activity status. The differences in the key spectral markers may be attributed to multiple factors. First, the 2 studies used different Raman systems. The *in vitro* study used a Raman microscope with a higher lateral (spatial) resolution than the fiber-optic probe used in the *in vivo* study. Second, the Raman microscope has an axial (depth) resolution of a few microns and primarily assessed the esophageal epithelium sampled in the mucosal biopsies. By contrast, the fiber-optic probe had a penetration depth of approximately 1 mm and a lateral resolution of approximately 1–2 mm. As such, the *in vivo* probe enabled us to capture biochemical changes from a larger volume of esophageal tissue extending from the superficial epithelium through to the deeper layers including the lamina propria. Last, differences in tissue state (*in vitro* vs *in vivo*) may also have contributed to the observed differences in the key Raman spectral peak locations.

A novel aspect of our study is the observation that the lipids (at 1,076/1,128 cm^−1^ and 1,303/1,174 cm^−1^) and protein (at 1,003/1,056 cm^−1^) content were significantly different between the proximal and distal esophageal sites in children with aEoE, but not in children with iEoE. These findings, for the first time, provide objective evidence that the biochemical composition can be uneven across the esophagus in active EoE, and it can normalize with adequate control of the disease activity. This observation is consistent with our current understanding that EoE is a patchy disease based on the variable distribution of the intraepithelial eosinophils and an uneven involvement of EoE-relevant esophageal epithelial features and lamina propria (35).

Although our study identified unique Raman intensities with biological basis and statistical significance, there are still several important limitations and avenues for future research. One limitation of our study was the relatively small sample size, which limited the external validity as well as our ability to conduct more detailed analysis. Additional study groups, such as children with GERD, could be included as a separate comparison group to distinguish between allergen-mediated inflammation in EoE and gastroduodenal refluxate-mediated inflammation in GERD. Investigating the relationship between key spectral markers and each of the components of EoEHSS would provide a more comprehensive understanding of the intricate relationship between the tissue-level biochemical and architectural changes in EoE. Another important area of research would be to explore the impact of EoE therapy (such as proton-pump inhibitor, topical steroids, or biologics) on the *in vivo* esophageal Raman spectra. Another limitation to consider is that our non-EoE control group was entirely comprised of females. Although limited research exists on the impact of sex on *in vivo* Raman spectra in esophagus, previous studies have found sex-specific differences in the *in vivo* Raman spectra acquired from colorectal tissue of healthy volunteers (36). As such, it is possible that sex may also have an impact on the *in vivo* Raman spectra in the esophagus. Lastly, this was a single-center study involving only pediatric patients. A prospective multicenter study involving all ages is needed to provide a more robust evaluation of the diagnostic and prognostic potential and to validate the applicability of this technology in clinical practice.

Our study has several strengths that enhance its significance and impact. It is the first study to demonstrate the ability of Raman spectroscopy to detect *in vivo* biochemical changes in the esophageal tissue during EGD in children. This innovative approach represents an important step forward in our ability to understand the real-time tissue biochemistry in EoE. We designed a customized fiber-based Raman probe compatible with the current white light endoscope used in pediatric endoscopy, which represents a significant technical advancement. An important strength of our study is our use of a standardized protocol that maintained consistent power and integration time for each measurement within and across participants. This allowed us to minimize measurement bias and ensure the accuracy and reproducibility of our results. Finally, an innovative aspect of our study was demonstrating that the portable Raman system combined with a pediatric endoscope-compatible fiber-optic Raman probe holds the potential to emerge as a non–tissue-obtrusive based approach for real-time identification of EoE (37,38). This can provide a significant advantage over traditional methods that often require invasive tissue biopsies. Realizing this potential could reduce the delay in diagnosis, foster timely, and effective management of EoE.

In conclusion, using a portable Raman system coupled with a pediatric endoscope-compatible fiber-optic Raman probe, we identified unique spectral markers reflective of the biochemical composition of the esophagus in children affected by EoE. This innovative approach can advance our understanding of the pathobiology of EoE, help to reduce delay in diagnosis of EoE, and potentially obviate the burden associated with collecting and examining multiple esophageal biopsies.

## CONFLICTS OF INTEREST

**Guarantor of the article:** Girish Hiremath, MD, MPH.

**Specific author contributions:** A.L. contributed to conceptualizing the study, acquisition, analysis, and interpretation of data, drafting the manuscript, and critical revision of the manuscript. E.H. contributed to the acquisition, analysis, and interpretation of the data and made critical revisions to the manuscript. G.T. contributed to conceptualizing the study and critical revision of the manuscript and technical support. H.C. contributed to conceptualizing the study, analysis, and interpretation of pathological data and made critical revisions to the manuscript. E.S.D. contributed to conceptualizing the study and interpretation of data and made critical revisions to the manuscript. A.M-J contributed to conceptualizing the study, acquiring, analyzing, and interpreting data, making critical revisions to the manuscript, obtaining funding, administrative, technical, and material support, and study supervision. G.H. contributed to conceptualizing the study, acquisition, analysis, and interpretation of data, initial draft and made critical revisions of the manuscript, and obtained funding, administrative, technical, and material support. Each of the authors has approved the final draft submitted.

**Financial support:** A.L. is supported by the Vanderbilt's Academic Pathways Postdoctoral Fellowship. G.T. is supported by the National Institute of Health (NIH) R01CA212147. A.M.J. is supported by the National Institutes of Health (NIH) R01CA212147 and R01HD081121. G.H. is supported by the American College of Gastroenterology Junior Faculty Development Award and the NIH 1K23DK131341. The content is solely the responsibility of the authors and does not necessarily represent the official views of the NIH.

**Potential competing interests:** G.H. serves a consultant to Bristol Myer Squibb, Regeneron, and Sanofi. In addition, he has received speaker fees from Bristol Myer Squibb. All other authors declare that the research was conducted without commercial or financial relationships that could be construed as a potential conflict of interest.

**Study Highlights**

**WHAT IS KNOWN**

✓ Eosinophilic esophagitis (EoE) is a debilitating, immune-mediated inflammatory condition of the esophagus affecting all ages.
✓ The biochemical composition of the esophagus in physiologic state affected by pediatric EoE has not been studied.

**WHAT IS NEW HERE**

✓ We developed a pediatric endoscope-compatible Raman spectroscopy probe to profile *in vivo* biochemical composition of the esophageal tissue in children during esophagogastroduodenoscopy.
✓ Our results indicate that the Raman intensities reflective of esophageal lipid and protein content accurately distinguish children with and without EoE and children with active from inactive EoE.
